# Death and disappearance: Measuring racial disparities in mortality and life expectancy among people in state prisons, United States 2000–2014

**DOI:** 10.1371/journal.pone.0314197

**Published:** 2025-02-06

**Authors:** Bryan L. Sykes, Ernest K. Chavez, Justin D. Strong

**Affiliations:** 1 Jeb E. Brooks School of Public Policy & Department of Sociology, Cornell University, Ithaca, New York, United States of America; 2 Department of Criminology, Criminal Justice and Emergency Management, California State University, Long Beach, Long Beach, California, United States of America; 3 Department of Justice Studies, San Jose State University, San Jose, California, United States of America; University of North Carolina at Chapel Hill, UNITED STATES OF AMERICA

## Abstract

**Background:**

Research on carceral institutions and mortality finds that people in prisons and jails have a high risk of death immediately following release from custody and that while incarcerated, racial disparities in prisoner mortality counter observed death patterns among similarly situated non-incarcerated, demographic groups. Yet, many of these studies rely on data prior to the millennium, during the COVID-19 pandemic, or are relegated to a small number or select group of states. In this paper, we explore changes in mortality and life-expectancy among different demographic groups, before and after the Great Recession, across forty-four states that reported deaths in custody to the federal government between 2000 and 2014.

**Methods:**

Drawing on a novel dataset created and curated, we calculate standard, age- specific quantities (death rates and life-expectancy) using period lifetable methods, disaggregated by race and sex, across three different periods (2000–2004, 2005–2009, and 2010–2014) for each state. Ordinary least squares regression models with state and year fixed-effects are included to examine state-level factors that may explain differences in prisoner mortality rates between 2000 and 2014. We also benchmark death counts reported to federal agencies with official state reports to cross-validate general mortality patterns.

**Results:**

Among imprisoned men, age-specific trends in mortality have shifted across the three periods. Following the Great Recession and the push for criminal justice reforms, prisoner mortality dropped significantly and is concentrated at older ages among men during 2010–2014; the shifting pattern of mortality means that men age 30 in 2010–2014 had similar death rates as men in their early 20s during 2000–2004, representing a 7.5 year shift in age-specific mortality rates. Gains in the mortality decline were disproportionately experienced by Non-Hispanic White and Non-Hispanic Black men, with the latter experiencing the greatest gains in life-expectancy of any demographic group. State-level violent crime rates are strongly and positively associated with prison mortality rates across states, net of socioeconomic and political factors. The large and significant disappearance of deaths in prisons from official data reported to federal agencies calls into question the narrowing gap in racial disparities among people in carceral facilities.

**Conclusions:**

Legal decisions and social policies aimed at reducing mortality may be most effective in the short-run; however, the effects of these policy changes may fadeout over time. Research should clearly discern whether changes in mortality rates across states are due to diminished gains in social policies or increases in the disappearance (or underreporting) of deaths in custody. Understanding how and why gains in survivorship may stall is important for aligning health initiatives with social policy to facilitate maximal and consistent mortality declines for all demographic groups.

## Introduction

The United States has experienced tremendous growth in incarceration since the mid-to-late twentieth century [1]. A robust body of scholarship demonstrates how the growing carceral state continues to be linked to other racialized institutions of confinement and control [2–4]. Consequently, experiencing incarceration holds important implications for Black men and women, whose life-course trajectories are disproportionately affected by imprisonment, particularly in household sample surveys where they significantly “disappear” from both public life and official indicators of social inequality [5–9]. Research shows that while racial disparities in incarceration have declined slightly over the past decade [10], the persistent consequences of incarceration remain [9,11]. Racially disparate health consequences extend not only to formerly incarcerated individuals but also to their families and communities [12–16], thereby reproducing social and racial inequality.

The onset of the Covid-19 pandemic during 2020 brought renewed public scrutiny to both demographic disparities in healthcare and the state of medical provisions inside carceral facilities. Despite the U.S. Supreme Court acknowledging, as early as 2011, that overcrowding and inadequate medical care create deadly prison conditions [17–22], the carceral state remained largely unchanged in its approach to prisoner healthcare. The pandemic highlighted circumstances under which millions of people living behind bars are made disproportionately vulnerable to infection, morbidity, and death [23], with research showing that the mortality of people in carceral facilities increased substantially during the pandemic [24]. Furthermore, there is evidence to suggest that local, state, and national statistics on infections and deaths were undercounted [25,26], with some states deliberately withholding data on death counts associated with the pandemic [27].

A similar controversy surrounding carceral facilities unfolded in September 2022 when the U.S. Senate Subcommittee on Investigations exposed data inaccuracies on prison death. The Department of Justice (DOJ) was undercounting prison and jail mortality, while also failing to collect demographic and circumstantial data on in-custody mortality [28,29]. The Subcommittee states that at least 990 in-custody deaths—341 of which occurred in state prisons—went unreported in 2021 alone. According to The Subcommittee’s report, undercounting occurred because the Department of Justice failed, in part, to implement the Death in Custody Reporting Act of 2013 (DCRA). In fact, the Subcommittee’s report states that as early as 2018, the Office of the Inspector General (OIG) warned that the DOJ’s method of implementing DCRA could lead to unreliable, inaccurate, and incomplete data. In this way, the pandemic did not create a health crisis inside America’s prisons, but rather reveals the consequences of preexisting health implications and data inaccuracies of mass incarceration that have been litigated before the U.S. Supreme Court [17].

In this article, we examine how patterns of mortality and life-expectancy in prisons have changed since the turn of the millennium and prior to the COVID-19 pandemic. Our study contributes to a growing body of research on the invisibility, hiddenness, and disappearance of incarcerated people from federally collected data. In doing so, we make four important contributions to the literature. First, we document the changing patterns of mortality within prisons by race, sex, and state since 2000, while also exploring whether observed death counts in national data match official reports to federal and state courts overseeing healthcare provisions in select states. Second, we present a new method for estimating the population distribution used to construct race- and sex- specific denominators for mortality rates among people in prisons and jails. Third, we document how state-level factors are associated with levels of prisoner mortality over time. Lastly, we draw attention to pressing questions raised by scholars, researchers, and advocates about the medical capacities of state prisons. We conclude with a detailed discussion about California and Arizona—two states that continue to struggle with court-mandated reform efforts—in order to illustrate the persistence of health disparities in mortality among people in prisons despite the shifting criminal justice landscape.

### Incarceration, premature mortality, and racial disparities

A growing body of research documents the consequences of incarceration and contact with the criminal legal system on the health of individuals and populations [30,31]. In a study on the mortality rates of New York state parolees, Evelyn Patterson found that each year of incarceration was associated with a 15.6% increase in the odds of dying, and that one year of time served in prison diminished life-expectancy by two years across a 1989–1993 parole cohort [12]. Another study of a 1999–2003 Washington state parole cohort produced similar findings, wherein the risk of death among the formerly incarcerated was 3.5 times the level of state residents who had not been incarcerated [32]. Risk of death was especially pronounced during the first two weeks of parole, during which the paroled individual was 12.5 times more likely to perish [32]. Such mortality risks are accentuated by housing and employment instability, strained social and familial ties, and access to high risk activities, such as intravenous and opiate substance use [31–35]. While several studies have focused on health risks of paroled populations, few have explored probation and juvenile detention. One study shows that the mortality rates of persons on probation are 2.1 times greater than the general population, over three times higher than people held in jail, and 2.81 times higher than people held in prisons [36]. Similarly, juveniles exposed to detention also experienced greater mortality risks as adults [37].

The negative impact of incarceration on premature mortality is not specific to the U.S., as formerly incarcerated men in Russia have a mortality rate roughly double that of the non-incarcerated population [38]. Similarly, healthcare utilization by current and former prisoners in Canada rely on a range of medical services, compared to the general population, suggesting another link between incarceration and heightened morbidity [39]. These studies illustrate that incarceration, indeed, affects a person’s health and well-being, and that the post-release period is a particularly vulnerable time for former prisoners.

While incarceration negatively affects health of incarcerated people in general, prison mortality rates are also stratified by race. Patterson’s research demonstrates that incarceration increases the death rate for white men but lowers it for black men [40]. This difference can be attributed to factors outside of the prison system, such as deaths caused by firearms, vehicular accidents, or other aspects of racialized social environments. Similar findings have been observed using different data and methods [41]. Thus, outside of prison, African Americans experience disproportionately higher mortality rates than that of whites [42]. These findings do not suggest that incarceration is “healthy,” but rather, Patterson’s study reflects the severity of broader social inequality in many African American communities.

The high rate of premature mortality among African Americans is driven, in part, by homicide [43]. Although homicide has consistently remained a leading cause of death for African Americans, and is understood as constituting a public health crisis, Black homicide rates have received neither adequate attention nor sufficient resources to redress it [43,44]. The crime decline–the precipitous and unexpected drop in homicide and violent crime beginning in the early 1990s –resulted in a roughly 50% decline in the rate of homicide [43]. According to Sharkey and Friedson’s research, this decline in homicide was associated with a .8-year increase in life-expectancy for Black men, and a reduction of 1,156 per 100,000 in years of potential life lost [43]. Thus, reducing homicide is likely to decrease the gap in life expectancy between racial groups, and yet, few resources have been allocated toward this goal. Between incarceration and early deaths, African American men are disappearing and missing from society at an alarming rate, the effects of which are experienced beyond the individual in families and communities [43–46]. In fact, incarceration is a significant driver of inequality that disrupts the social organization, cohesion, and control necessary for communities to address their collective needs and to mobilize resources [47–49].

Finally, studying health disparities among prisoners also highlights an important epistemological challenge. Similar to Bruce Western’s findings [50]–regarding how national statistics on employment exclude incarcerated populations and thus create biased findings–other demographic research shows that that the exclusion of prisoners from national health surveys obscures population data on the causes and consequences of health disparities [51]. Given incarcerated people tend to exhibit worse health profiles compared to the general population, their inclusion in national health surveys would likely lower the overall estimation of population health in the U.S. [51]. Because African-Americans are incarcerated at significantly higher rates than other racial groups [9,11,15], the underreporting of Blacks from national surveys more than likely results in an under-estimation of racial health disparities [8].

### The health of incarcerated people and healthcare quality in prisons

Healthcare services within carceral facilities are also important to understanding in-custody mortality rates. Roughly half of all prisoners self-report a chronic medical condition, most often noting asthma, diabetes, hypertension, cancer, visual impairment/blindness, deafness, and obesity [52–54]. Those with a history of incarceration are also more likely to suffer from infectious diseases [55]. The most common communicable diseases that incarcerated people are prone to contracting include HIV and AIDS, syphilis, gonorrhea, chlamydia, trichomoniasis, tuberculosis, hepatitis B and C [52]. The leading causes of death in prison are heart disease, cancer, liver disease, and AIDS and the vast majority of these deaths are precipitated by social conditions that precede incarceration [56]. Furthermore, prisoners are more likely to lack medical insurance prior to incarceration or to have their Medicaid coverage suspended or terminated as a result of going to prison. Not only do prisoners present significant health needs, but they also belong to populations that routinely underutilize or lack access to healthcare [31]. While the passing of the Affordable Care Act (ACA) has significantly improved the insurance coverage of formerly incarcerated men, this coverage has not translated to increased health service utilization [57–59].

Although variation exists across policies and practices across states, medical testing and screening procedures are generally under-utilized inside prisons, particularly for sexually transmitted infections [60]. While improved screening practices could potentially limit the spread of disease and illness, the reverse is also true: carceral institutions can worsen health conditions and exacerbate disparities and social stratification [60–66]. For example, some states screen only incoming prisoners for medical conditions, while others only screen at the time of release, or while in custody [60,67,68]. Although research shows that healthcare screening and treatment during parole and probation supervision helps to reduce recidivism as well as the spread of infectious diseases, especially amongst Black, Latino, and low-income communities [69], few states provide screenings at both admission and release periods. Inadequate screening practices can also contribute to the spread of infectious diseases into other carceral and non-carceral populations, as has been the case with HIV and AIDS [60,70,71]. Romantic partners and family members for example can be exposed to illnesses upon release [72]. In this sense, carefully laid out screening and healthcare provisions for incarcerated populations could reduce the overall spread of disease [73,74]. Thus, as Sykes and Piquero observe, “Prisons and jails can help to ameliorate health inequalities depending on their policies, the state, how policies are implemented, and when inmates are exposed to them. Yet… [they] can also re-create the very health disparities that plague an inmate’s sending community” (p. 216) [60].

Beyond screening practices, inadequate healthcare can have other serious impacts on the well-being of an incarcerated person and their familial network. For women, having an incarcerated family member increases their risk for heart disease, stroke, and obesity [75]. Moreover, women with incarcerated husbands are more likely to experience deterioration in their mental health [76]. The health of children is especially impacted by parental incarceration. In studies conducted in both the U.S. and Denmark, infants and children are shown to have higher mortality risks when a parent is incarcerated [77,78]. Children of an incarcerated parent also tend to experience harsh psycho-social environments that can lead to harmful long-term mental health and behavioral patterns such as increased aggression [79–82]. The social disadvantage of having a parent incarcerated heightens the risk of delinquent behavior while also negatively impacting health, wealth, and adult levels of criminal activity [83]. Having a parent incarcerated at a young age can lead to learning disabilities, attention deficit disorders, developmental delays, and speech problems [82]. The overall effect of imprisonment on health extends far beyond the individual.

### Mortality and aging in prison

The growing population of aging incarcerated people is another important site to consider in the study of health and survivorship within prisons. Carceral facilities have lagged in providing appropriate care for chronic illness which has been rising in part, due to an increase in elderly prison populations [84]. While there is no standardized definition for what designates a prisoner as “elderly” [52], people who are confined to carceral facilities tend to age physiologically faster than the general population [52,84–86]. The National Commission on Correctional Health Care cites a 2017 report stating that elderly and aging prisoners comprise one of the fastest growing demographic groups in prison [87]. Nationwide, the number of incarcerated people aged 55 or older grew by some 400% between 1993 and 2013 [88]. In California, from 2015 to 2018, the number of prisoners 55 and older increased from 12.5% to 15% (16,212 to 19,389) of the state’s prison population [89]. Growth continues even though a plethora of research demonstrates that individuals tend to “age out” of crime as they approach their late twenties [90–94].

A few key penological drivers behind the growth of elderly prisoner populations includes the routinization of life, life without parole, and other long-term prison sentences [20,95]; but also, the replacement of judicial discretion with mandatory minimum sentencing, truth-in-sentencing laws, determinate sentencing, supplemented by a rise in prosecutorial discretion [52,96–98]. For example, in the state of California, Three Strikes legislation contributed to an unprecedented level of long-term sentencing and prison overcrowding at the turn of the millennium, which in turn worsened the provision and delivery of medical treatment behind bars [97]. Long-term incarceration also raises fiscal costs because prisons are responsible for providing a constitutionally adequate level of healthcare. Healthcare spending varies greatly by state and broaches broader issues such as quality of care, appropriate staffing, regional health differences, and cost-effective programming [99]. California’s prisoner healthcare costs are the highest in the nation at $19,796 per incarcerated person per year in 2015, which was three times the national average and up 25% from 2010 expenditures [46]. At the other end of the spectrum is Louisiana, which spends only $2,173 per incarcerated person per year [100]. The aging prisoner population is often a focal point of healthcare policy discussions because of the economic strain involved [101]. Unlike younger incarcerated people who tend to experience disease through acute and single episodes generally accompanied by relatively fast recovery, elderly prisoners often experience disease as a chronic and progressive process accompanied by slow recovery [90].

However, explaining the rise in aging prisoner populations is an ongoing subject of debate. While many researchers attribute this population growth to harsh sentencing laws enacted during the 20^th^ century, which lengthened time spent in prison, recent demographic scholarship has begun to challenge this assumption. A 2019 study suggests that the “crack down hypothesis” needs to be nuanced between a period effect and a cohort effect [102]. While a period effect would support the hypothesis that punitive sentencing laws affected all prisoners rather indiscriminately, a cohort effect would suggest that certain prisoner cohorts were disproportionately impacted by such policies. The authors explain their findings, stating, “Essentially, the younger birth cohorts from the 1980’s (e.g. those born in the 1960s) seem to have a high incarceration rate across periods even at older ages,” due to higher reported drug usage (p. 49) [102]. Accordingly, the increase in aging prisoner populations then is not so much an effect of harsh sentencing laws, but rather can be attributed to increases in prison admissions of birth cohorts that came of age during the 1980s.

One trend that remains clear, however, is the continued racialization of incarcerated people even amongst the aging. Just as the rise of the nation’s carceral population has been stratified by race, the same is true of today’s elderly prisoner population. According to a 2016 Bureau of Justice Statistics report on aging prisoner populations, the number of African American prisoners, age 55 or older, increased by 164% between 2003 and 2013 (from 16,100 to 42,500), far more than Whites or Latinos [88]. The rate of incarceration for African American prisoners, ages 40–54, increased from 880 per 100,000 to 1,925 during 1993–2003, and then up to 1,868 per 100,000 from 2003–2013. For African American prisoners, ages 55 and older, the rate grew from 174 per 100,000 to 284 between 1993–2003, and then up to 509 per 100,000 by 2013 [88]. Thus, the overrepresentation of African Americans in the nation’s prison system remains prevalent even amongst aging prisoners.

## Theoretical framework: Biopolitics, invisible populations, and decarceration

The undercounting of prisoner mortality by the Department of Justice raises critical concerns for researching shifts in the life expectancy and survivorship of incarcerated populations. There are three theoretical impacts of underreported deaths-in-custody on observed patterns of racial disparities internal to prison mortality to be considered here: Inaccurate demographic knowledge, the disappearance and exclusion of vulnerable populations, and the failure of legal compliance to improve mortality through prison reform. Each aspect is crucial toward understanding racial disparities in life expectancy and survivorship behind bars.

First, underreporting of death among vulnerable groups obfuscates biopolitical knowledge—that is, the recognition of normative patterns and irregularities among a given population—and therein thwarts the governmental capacity to intervene and offer potential correctives in the effort to extend and make productive use of life. Michel Foucault [103] describes biopolitics as a governmental strategy wherein the nation-state meticulously analyzes the various social, political, and economic conditions that affect a population’s health and well-being, typically through the use of demographic reports. Biopolitical governance relies on three aspects of population knowledge: (1) the political dynamics affecting states and their populations, (2) the impact of economic shifts, and (3) the potential threats to a population caused by diseases, pandemics, and endemics. A key function of biopolitical data here is the ability of the state to track changes in mortality and natality rates as a means to govern a population in particular ways (p. 354) [103]. However, if state-generated reports are systemically incomplete in terms of how death is being counted (or not), how might this reflect back on the state’s legitimate or illegitimate administration of social life? (p. 235) [104]. In the present-day context, the failure of the Department of Justice to accurately report in-custody mortality obscures official data and knowledge of both prison death and the racial disparities internal to life expectancy behind bars. Thus, incomplete data and the systematic underreporting of prison mortality not only problematizes the utility of biopolitical knowledge but speaks to the social and political determinations by which some lives are valued less than others.

Second, there is reason to believe that the underreporting of deaths in carceral facilities is consistent with existing research on the exclusion of people in prisons and jails from national statistics. Sociologists of inequality have identified how the exclusion and underreporting of incarcerated populations within household sample surveys, such as the Current Population Survey (CPS), and in official counts of the population, such as the American Community Survey (ACS), distorts and skews the accuracy of demographic data [8,13,105]. This exclusion has been shown to artificially inflate employment rates and other social markers related to the well-being of Blacks and Latinos, thereby masking inequality and perpetuating a myth of racial progress [8]. For instance, in *Invisible Men*, Becky Pettit [8] writes that this practice of exclusion misinforms both social policy and social science research because it “clouds our understanding of the American economic, political, and social condition” (p. vi). In other words, making vulnerable groups “invisible” within population reports not only obscures estimates of social disparities, but it also severely undercuts the ability of social science to mitigate or alleviate racial disparities in morbidity and mortality.

Third, there is reason to believe the underreporting of deaths in custody may be tied to historical periods of economic distress and restructuring of the criminal legal system. The Great Recession provided a reorganizing jolt to the politics, debates, and legal issues surrounding mass incarceration [106,107]. Since the Great Recession began in December of 2007 [108], the discourse of mass incarceration has tilted towards an embrace of modest institutional reform as state political actors could no longer justify the high costs of incarceration in the context of economic turmoil. The Great Recession is also tied to the Supreme Court’s *Plata v*. *Brown* decision, which mandated that the state of California decrease its prisoner population. It is, therefore, critical to understand how prison systems translate political, economic, and legal challenges into bureaucratic policies, incentives, and outcomes that are defined and assessed accordingly. [109,110]. For the present study, the Great Recession provides a temporal marker for studying the extent and impact of prison reforms, including trends in mortality among people in state prisons. Improvements in mortality trends across demographic groups, then, would be consistent with the decarceration movement following the Great Recession; however, an alternative explanation that is consistent with the aforementioned theoretical rationale is that mortality improvements across racial groups is not due to prison decarceration but rather the undercounting of the deaths of incarcerated people in official statistics.

Our current study investigates how prisoner mortality and life-expectancy have changed in the new millennium, particularly following a series of reforms to the criminal legal system that have led to declines in the number of inmates and the expansion of medical previsions for incarcerated people. While previous research by Patterson examined prisoner mortality patterns, that study was confined to 1985–1998, a period prior to the peak of mass incarceration and significant policy reforms across states [12]. Other research has focused on the mortality risk of prisoners once paroled [12,32] and the impact of the COVID-19 pandemic on mortality in carceral facilities [111]. There is no study, however, that has examined mortality levels in prison facilities immediately before and after the Great Recession but prior to the pandemic, during distinct periods of carceral growth and retrenchment. Our article fills this gap by focusing on how patterns of mortality and life-expectancy have changed across race, sex, and state since 2000, and whether state-level criminal justice and socioeconomic factors explain the observed variation in mortality rates among prisoners. We explore whether the underreporting of deaths in custody is a potential explanation for observed reductions in racial inequality among different demographic groups of people in prisons.

## Data

We leverage data from multiple sources to examine how prisoner mortality has changed in the wake of the Great Recession and the criminal justice reform. First, we use data from the National Corrections Reporting Program (NCRP). These data are collected by the U.S. Census Bureau and Abt Associates, on behalf of the U.S. Department of Justice’s Bureau of Justice Statistics (BJS). The NCRP compiles individual-level data on admissions, releases, post-confinement community supervision and yearend prison custody records. The data are routinely used to address policy related matters regarding the prison population’s size and composition, levels of recidivism, and reentry experiences among parolees and individuals released from custody. For the purposes of our paper, we leverage information on the deaths of incarcerated people in state prison facilities by age, sex, and race for all reporting states from 2000 through 2014.

A second source of data were obtained from the Uniform Crime Reports (UCR), collected by the Federal Bureau of Investigation (FBI). We draw on composite measures of violent and property crime by state and year to examine how external criminological conditions are associated with prisoner mortality. This approach would inform our ability to assess how potential admissions (i.e., an increasing rate of violent vs. non-violent prisoners) may affect the underlying prisoner mortality rates within a state.

UCR crime data have several issues that should be noted. First, the UCR does not include all crimes experienced in the population. Many crimes are unreported, necessitating the collection of data on population victimization. Second, police departments may also report crime data inconsistently or inaccurately to the FBI, complicating official measures of specific crimes. Nevertheless, the UCR data are the routinely used to track crime rates.

Third, state imprisonment rates were obtained from the Bureau of Justice Statistics (BJS). The BJS’s Corrections Statistical Analysis Tool (CSAT) allows for the extraction of the number of prisoners under state or federal jurisdiction sentenced to more than 1 year per 100,000 U.S. residents. Year-end population, admissions, or releases for each state-year can also be extracted from CSTAT.

Lastly, we append to our dataset state-level economic and political data from the University of Kentucky Center for Poverty Research (UKCPR). The UKCPR Welfare Data contain economic and political indicators by state and year, including the population counts, poverty rates, the unemployment rate, and whether the executive branch of state government is headed by a democrat or republican. We use these data as controls in our models.

## Measures

The NCRP admission, release, and custody files contain different causes of death that we include in the numerator of our mortality rates: deaths due to natural causes, suicides, homicides, executions, other deaths, and accidental injuries. We leverage prisoner birthdate and the reporting date for each prisoner file to construct the age of the person for the release and custody files. Unfortunately, there was not a reporting date in the admission file, so we used the admission date in lieu of the reporting date to construct the ages of people in prison. Race was coded based on a combination of race and ethnic questions, resulting in our construction of Non-Hispanic Whites, Non-Hispanic Blacks, and Hispanic racial categories. It is worth noting that the use of “Hispanic/Non-Hispanic” as an ethnic distinction was differentially adopted by state correctional facilities overtime, and it is unknown when each state began collecting ethnicity as a race-based measure; however, the NCRP data contains this identifier at the person-level. Sex was reported as either male or female.

We also rely on various measures from the UCR, CSTAT and UKCPR data. Violent and property crimes represent the number of offenses of each type per 100,000 residents, and the imprisonment rate is based on the number of people in prisoner per 100,000 residents. Poverty and unemployment rates capture the economic conditions of each state. We also code whether the executive branch of the state is headed by a democratic governor. S1 Table, presents our operationalizations and descriptive statistics for these measures.

## Methods

We employ standard demographic methods to construct age-specific death rates and life-expectancies. By combining the number of deaths (nDx) and the mid-period counts of inmates (nKx) between age (x) and x+n (where n is the length of the age interval), we can compute period age-specific death rates (nMx) for each age group for a single year (T), as displayed in Eq 1.


$$nMx(T)=\frac{nDx(T)}{\left(nKx)(T\right)}$$ (1)

In some analyses presented, estimates of death rates are based on for 5-year intervals. In such instances, the denominator uses the average mid-period population count based on the start and end points of the period. Wachter shows that standard life-table entries (probabilities of dying, survivorship, life-expectancy at age x (e_x_), etc.) can be computed once period, age-specific mortality rates have been calculated [112]. We compute age-specific mortality rates (nMx) and life-expectancy at age 20 (e_20_) for all prisoners, by sex and across three periods: 2000–2004, 2005–2009, and 2010–2014. We also disaggregate these trends by race.

While scholarship on social inequality in health due to incarceration faces a number of data and methodological challenges [113], tracking the impact of the prison system on life-expectancy is important for understanding the effects of incarceration on current and future population health. Unfortunately, federal prison statistics do not provide the age and race distributions for the prison population. Largely owing to data limitations and availability, Patterson draws on the Census of State and Federal Adult Correctional Facilities (CSFACF) and the Surveys of Inmates in State and Federal Correctional Facilities (SISFCF) to estimate the size and distribution of prisoners at the regional and state level, respectively, under a set of backward deduction assumptions [40]. While this approach is reasonable given the significant data limitations and availability at the time of her analysis, this method is of limited utility for our study for several reasons. First, the SISFCF and CSFACF data have not been collected and/or updated since 2004 and 2005, respectively. Ten of the fifteen years in our study occur after these data were collected. While scholars normally assume an unchanging correctional population distribution by race, sex, age, and education in the years following the collection of SISFCF data [8,9,113–115] and apply BJS counts of the carceral population to the SISFCF distributions, this methodological approach is not feasible for the study of mortality because the SISFCF sampling frame does not include deceased prisoners. Thus, the lack of recent population counts from CSFACF and the lack of recent survey measures in SISFCF, as well as the potential sampling biases in the SISFCF data, render this method of constructing a prison population distribution potentially problematic.

Second, recent changes to the NCRP data files allow for the construction of age-specific population counts by race, sex, and period for all admissions, releases, and people in custody. However, because the admission, release, and custody files are often missing crucial information, particularly about when a prisoner’s individual-level file was received by the federal government–which is critical to obtaining reliable annual counts of imprisoned people and deaths–and because our analyses also use state-level characteristics to examine factors that explain mortality in prisons, we develop a new method to construct age, sex, and race-specific population counts for the denominator of the mortality rate.

We marry data on the state resident population from the UKCPR, state imprisonment rates (per 100,000 residents) from the BJS CSTAT online portal, and the age, sex, and race distribution from NCRP to obtain reliable counts of the prison population and its distribution. Specifically, we construct annual prison population counts by reverse engineering the imprisonment rate for each state using the CSTAT and UKCPR data, such that the prison population counts (*K*^*Prison*^) are the product of the imprisonment rate (IR) and the number of state residents (*K*^*Residents*^) divided by 100,000 (the initial per capita multiplier) for a particular year (T), as displayed in Eq 2.


$$K^{Prison}(T)=\frac{IR(T)*K^{Residents}(T)}{100,000}$$ (2)

Next, we generalize this approach to derive the sex, race, and age-specific prison population counts for a particular state. Specifically, we multiply the reliable prison population counts obtained from Eq 2 by the age, sex, and race specific distributions of prisoners in the NCRP, obviating the need to leverage the SISFCF data for these distributions and backwardly estimating prisoner population counts from region to state after applying the totals to the regional NCRP data, as Patterson [40] deftly demonstrates. Our method also minimizes potential sampling and measurement errors because we do not need to rely on periodic surveys of inmates in carceral facilities who may be present in the NCRP but absent in the sampling frame of the Surveys of Inmates; demographic shifts in the prison population within a calendar year and over time, or prisoner deaths during the interview period, would mean that some prisoners would not be included in the second-stage sampling frame of the SISFCF. Thus, this method ensures that the prison population at risk of death matches the observed death patterns with the NCRP data, and these data are now publicly available unlike at the time of Patterson [40].

Fourth, we examine whether and how the scale of imprisonment, crime, and other socioeconomic factors (political party in office, poverty rates, and unemployment rates) are associated with prisoner mortality across the states in our study. We construct a panel dataset and estimate a model of how logged mortality rates for state (s) during year (t) are associated with a vector of X demographic variables, as displayed in Eq 3.


$$\text{ln}\left(Mortality\;Rate_{st}\right)=\beta_{0}+\beta X_{st}+\gamma_{s}+\lambda_{t}+\varepsilon_{st}$$ (3)

State (*γ*_*s*_) and year fixed-effects (*λ*_*t*_) are included in the model to capture state-specific and period-specific events that are not directly measured in the regression equation (criminal justice spending, public beliefs about crime and mass incarceration, etc.). All standard errors are clustered on the state identifier.

Our modeling of prison mortality as a function of violent and property crime is important for two reasons. First, violent and property crime represent potential flows of individuals into the penal system. Depending on whether the violent or property offense is a felony or misdemeanor, felonies generally require longer periods of confinement. Therefore, higher rates of felony violent or property crime would mean that more people are at risk of imprisonment, which is distinct from the people who are captured in our imprisonment rate measure.

Second, there are qualitative reasons to include violent and property crime in our regression model. One such reason is that the people who commit violent and property crimes could be either victims or perpetrators of violence while imprisoned. Thus, higher rates of violent offenses in the state may mean that violence may increase within prisons, once arrested and convicted, leading to a potential increase in prisoner mortality. Additionally, higher crime and imprisonment rates would mean that the number of people at risk of dying in prison is also higher due to prison overcrowding and additional strains on the dispensary of healthcare behind bars.

Lastly, we use raw data generated at the state level as a spot check on the NCRP data. Here we check mortality data for three states, California, Arizona, and Texas. We have selected these states because all three prison systems have a particularly relevant history of healthcare litigation in which the preventable deaths of incarcerated people were of significant concern. All three states also present vastly different prison healthcare models: California’s is operated by a state agency but remains under federal receivership; Arizona’s is privatized and has been under extensive court supervision; and Texas’ is managed by health and medical schools within the state’s university system. Such different healthcare models not only affect the way in which prisoner morbidity and mortality are addressed, but each type of system may influence the institutional and administrative pathways by which such data are reported nationally. Finally, all three systems make state-level prison mortality data available in different outlets and methods. For California, data is drawn from the California Corrections and Health Care Services (CCHCS) annual Mortality Review Report [89,116]. The CCHCS receives mortality reports directly from prison medical administrators and is responsible for publishing an annual report that tracks all deaths. For Texas, prison mortalities are reported to the Texas Office of the Attorney General. The Texas Justice Initiative then makes monthly open record requests to obtain the raw data used to produce these reports and publishes them online [117]. For Arizona, all prisoner mortality data is collected and evaluated by the Arizona Department of Corrections, Rehabilitation and Reentry (ADCRR). Monthly reports are published on the ADCRR’s website and include yearly death totals for a 10-year period [118]. Checking the NCRP data against state level data will help ensure accuracy in our assessment of overall prison mortality trends.

## Results

**Fig 1:**
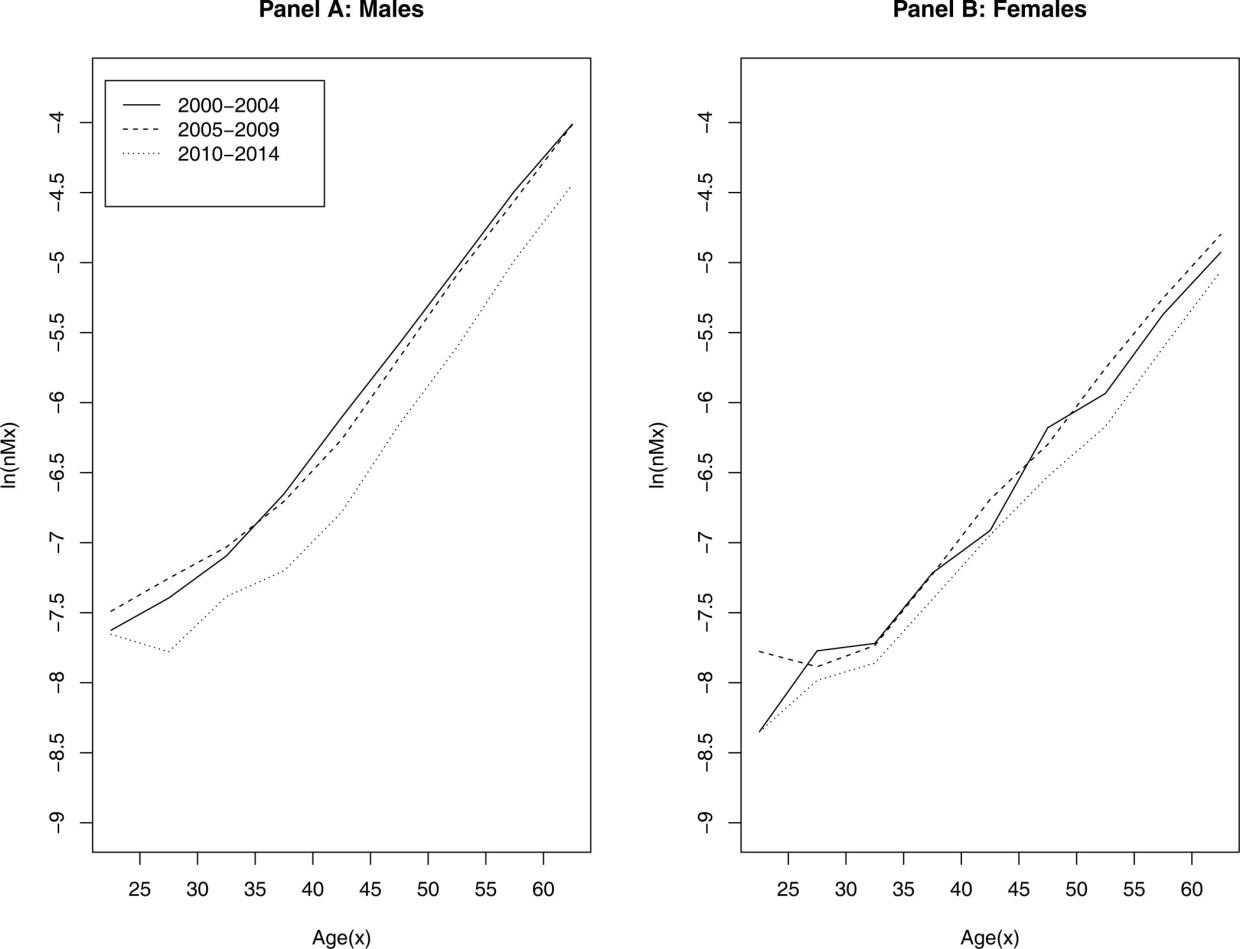
Age-specific mortality rates among state prisoners across periods by sex, 2000–2014.

Fig 1 displays the logged age-specific mortality rates (nMx) among state prisoners, by sex, across three periods in our study: 2000–2004 (the solid line), 2005–2009 (the dashed line), and 2010–2015 (the dotted line). Logged death rates reveal the underlying force of mortality (or the hazard of death) as a function of age. Demographic methods present log transformations of mortality rates to illustrate the increasing risk of death across the life-course, resulting in a linear, increasing function (see Wachter 2014, p. 66; Preston, Heuveline, and Guillot 2001, p. 51).

There are three main findings presented in this illustration. First, among male prisoners, age-specific trends in mortality have shifted across the three periods. Prisoners age 20–34 during the 2005–2009 period had slightly higher death rates than prisoners of the same age five years earlier (the 2000–2004 period). However, after age 35, the overall trend changes direction, with mortality falling, slightly, at older ages during 2005–2009.

The second main finding is that, following the Great Recession and the push for criminal justice reforms, prisoner mortality dropped significantly and is concentrated at older ages among incarcerated males during 2010–2014. These gains are extraordinary, as, for example, the general shift in the pattern of mortality means that men age 30 in 2010–2014 now have similar death rates as men in their early 20s, representing a 7.5 year shift in the age-specific mortality rates of male prisoners since the 2000–2004 period. The relative gains in survivorship decreases and narrows at older ages but the overall pattern persists.

The third main finding is that female prisoners also experienced a mortality decline by 2010–2014. However, their gains in survivorship are marked by a mortality deceleration at older ages, as the mortality trend (i.e., the dotted line for 2010–2014) widens after age 35, in comparison to the other two periods. Taken together, these trends suggest that survivorship and life-expectancy have improved since the end of the Great Recession.

**Fig 2:**
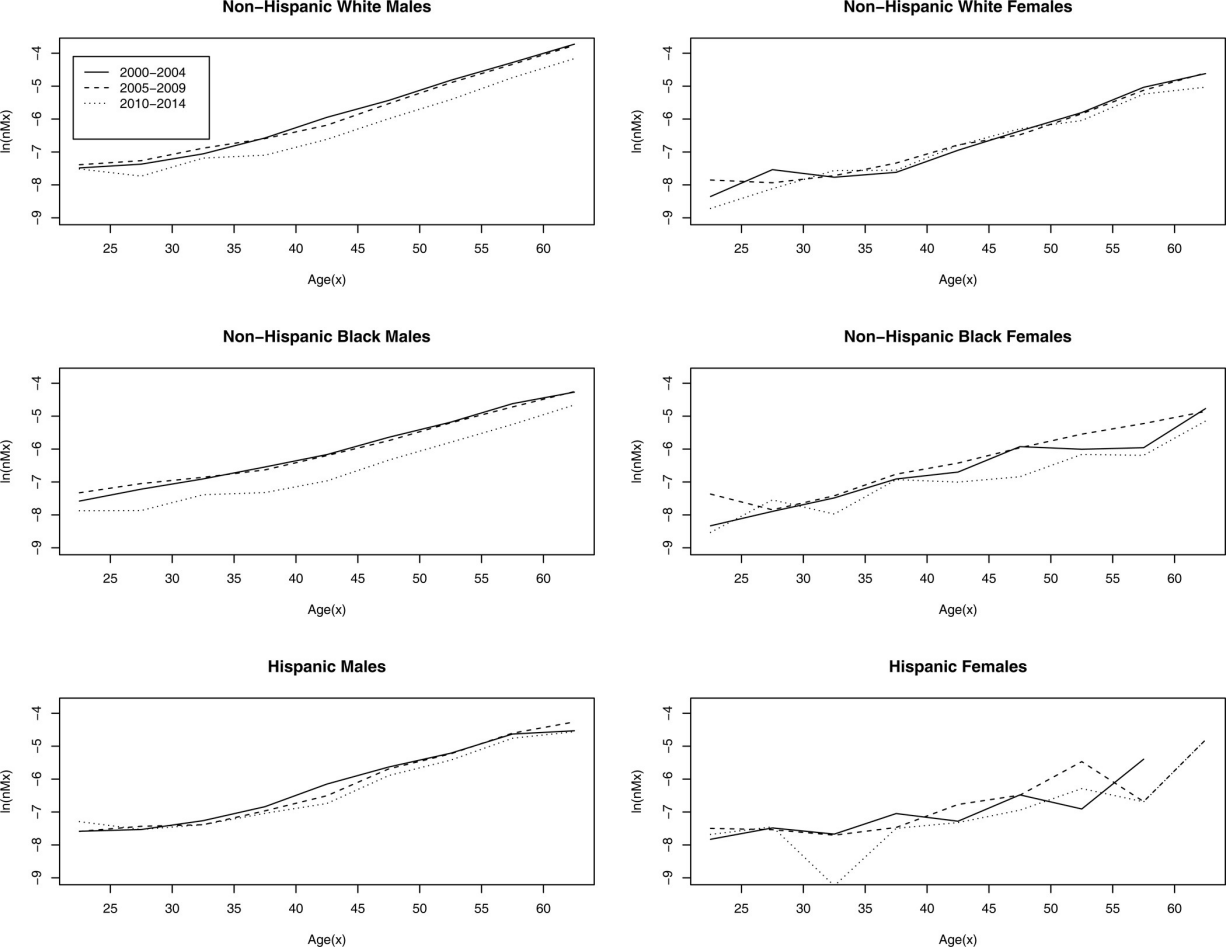
Age-specific mortality rates among state prisoners across periods, by race & sex, 2000–2014.

Next, we examine whether improvements in mortality are uniform across racial groups. Fig 2 shows the age-specific mortality rates among state prisoners, by race and sex, across the three periods. This figure clearly demonstrates two important observations. First, gains in the mortality decline are disproportionately experienced by Non-Hispanic White and Non-Hispanic Black men, with the latter experiencing the greatest gains of any demographic group. Hispanic men do not display marked changes in mortality across the three periods, except, perhaps, in their early-to-mid 40s. Thus, improvements in survivorship among Black men, and to a slightly lesser degree, white men, largely explain the overall pattern in Fig 1.

Second, the mortality deceleration at older ages for women is largely driven by improvements in survivorship among Non-Hispanic Black women. There is relatively little change in mortality for White women after age 30. Hispanic women display significant variation in their mortality patterns across the three periods.

To place into context how these declines and shifts in mortality have affected the underlying life-expectancy of prisoners, Table 1 presents the absolute changes in life-expectancy at age 20 among prisoners, by race and sex, across the three periods. This table also displays White-Black and White-Hispanic differences in these life-expectancy gains (or losses) between periods. There are three important findings in this table. First, male and female prisoners increased their life-expectancies by .76 and .17 of a year, respectively, over the 15-year period.

**Table 1:** Absolute changes in life expectancy at age 20 among people in prisons, by race & sex across periods, 2000–2014.

	2005–2009 vs. 2000–2004		2010–2014 vs. 2005–2009		2010–2014 vs. 2000–2004	
	Male	Female	Male	Female	Male	Female
**Total**	0.07	-0.10	0.69	0.27	0.76	0.17
**Non-Hispanic White**	0.11	0.00	0.75	0.13	0.86	0.13
**Non-Hispanic Black**	0.02	-0.32	0.84	0.58	0.86	0.26
**Hispanic**	0.07	-0.20	0.20	0.31	0.27	0.11
**White-Black Difference**	0.09	0.32	-0.09	-0.45	0.00	-0.13
**White-Hispanic Difference**	0.04	0.20	0.55	-0.18	0.59	0.02

Authors’ calculations from NCRP, UKCPR, and BJS data.

Second, declines in mortality rates substantially increased life-expectancy among White and Black prisoners. For example, compared to the 2005–2009 period, White and Black male prisoners increased their life-expectancies between three-quarters and five-sixths of a year during the 2010–2014 period (see the middle column). Black women, on the other hand, increased their life-expectancies by nearly three-fifths of a year during the same periods, in comparison to the modest (one-third a year) and moderate (one-eighth a year) gains among Hispanic and White women, respectively. The long-run, 15-year estimates show that White and Black men gained six-sevenths of a year in life-expectancy, compared to the one-eighth, one-quarter, and one-ninth gains among White, Black, and Hispanic women, respectively.

The final important finding from Table 1 is that within-sex racial differences persist or vanish over time. Gains in life-expectancy among White and Black male prisoners, for instance, disappear by the end of the 15-year period (see the last column), whereas gains in life-expectancy for Black women outpace those of White women by one-eighth of a year (i.e., -0.13). Additionally, gains among White male prisoners at the close of our time-series was greater than the gains by Hispanic men, by nearly three-fifths of a year.

We explore where these declines in prisoner mortality have taken place. Table 2 lists mortality rates per 10,000 prisoners and the relative change in prisoner mortality rates for forty-four states reporting to the NCRP. This table displays remarkable levels of, and changes in, mortality rates across states and periods. During the 2000–2004 period, the top ten states with the highest mortality were Kentucky (120.5), Florida (120.4), Colorado (119.2), Oregon (117.7), South Carolina (117.5), California (114.6), Pennsylvania (110.6), Tennessee (98.8), Georgia (98.6), and Missouri (98.6). Although mortality rates had fallen a decade later, only four of the original ten states remained in the top ten list during the 2010–2014 period: Kentucky (86.0, #1), California (60.8, remaining at #6), Georgia (56.4, climbing from a tie at #9 to #7), and Missouri (55.7, climbing from a tie at #9 to #8). Six states also did not report any deaths in custody over the three time periods: Delaware, Idaho, New Hampshire, New Mexico, Ohio, and Rhode Island.

**Table 2:** Mortality rates (per 10,000 prisoners) and the relative percentage change in prisoner mortality for forty-four states reporting to the NCRP, 2000–2014.

State	Prison Mortality Rates, 2000–2004	Prison Mortality Rates, 2005–2009	Prison Mortality Rates, 2010–2014	Percentage Change (2005–2009 vs. 2000–2004)	Percentage Change (2010–2014 vs. 2005–2009)	Percentage Change (2010–2014 vs. 2000–2004)
**Alabama**	36.6	80.2	24.9	119.1	-69.0	-32.0
**Alaska**	22.5	51.4	18.4	128.4	-64.2	-18.2
**Arizona**	88.7	89.2	51.1	0.6	-42.7	-42.4
**California**	114.6	83.1	60.8	-27.5	-26.8	-46.9
**Colorado**	119.2	80.4	46	-32.6	-42.8	-61.4
**Delaware**	0	0	0	-	-	-
**Florida**	120.4	80.5	47.9	-33.1	-40.5	-60.2
**Georgia**	98.6	83.2	56.4	-15.6	-32.2	-42.8
**Idaho**	0	0	0	-	-	-
**Illinois**	65.9	71.1	32.9	7.9	-53.7	-50.1
**Indiana**	95.6	94.9	50.7	-0.7	-46.6	-47.0
**Iowa**	38.6	73.6	32.3	90.7	-56.1	-16.3
**Kansas**	6.8	19.4	42	185.3	116.5	517.6
**Kentucky**	120.5	110	86	-8.7	-21.8	-28.6
**Maine**	0	4.6	36.9	-	702.2	-
**Maryland**	81.8	34.8	0	-57.5	-100	-100
**Massachusetts**	24.1	44.2	45.5	83.4	2.9	88.8
**Michigan**	75.5	54.2	21.8	-28.2	-59.8	-71.1
**Minnesota**	68.4	59.5	50.6	-13.0	-15.0	-26.0
**Mississippi**	9	12	23.2	33.3	93.3	157.8
**Missouri**	98.6	91.4	55.7	-7.3	-39.1	-43.5
**Montana**	30.2	22.5	70.9	-25.5	215.1	134.8
**Nebraska**	22.4	35.5	53.3	58.5	50.1	137.9
**Nevada**	62.3	64.8	67.3	4.0	3.9	8.0
**New Hampshire**	0	0	0	-	-	-
**New Jersey**	67.5	79	43	17	-45.6	-36.3
**New Mexico**	0	0	0	-	-	-
**New York**	93	65.7	40.1	-29.4	-39.0	-56.9
**North Carolina**	90.1	65.3	30.4	-27.5	-53.4	-66.3
**North Dakota**	33.8	34.4	19.4	1.8	-43.6	-42.6
**Ohio**	0	0	0	-	-	-
**Oklahoma**	80.5	24.6	25.6	-69.4	4.1	-68.2
**Oregon**	117.7	70.6	26.1	-40	-63.0	-77.8
**Pennsylvania**	110.6	77.5	17.2	-29.9	-77.8	-84.4
**Rhode Island**	0	0	0	-	-	-
**South Carolina**	117.5	104.4	35.6	-11.1	-65.9	-69.7
**South Dakota**	10.4	17.9	27.9	72.1	55.9	168.3
**Tennessee**	98.8	67.1	43.4	-32.1	-35.3	-56.1
**Texas**	60.6	83.5	48.4	37.8	-42.0	-20.1
**Utah**	83.6	75.6	66.2	-9.6	-12.4	-20.8
**Washington**	95.2	76.5	44.2	-19.6	-42.2	-53.6
**West Virginia**	91	85.3	68.5	-6.3	-19.7	-24.7
**Wisconsin**	89.6	56.2	39.8	-37.3	-29.2	-55.6
**Wyoming**	66.6	79.9	35.8	20	-55.2	-46.2

Note: Authors’ calculations using data from the NCRP, UKCPR, and BJS.

Table 2 also illustrates the percentage change in mortality between periods. Seven states–Kansas, South Dakota, Mississippi, Nebraska, Montana, Massachusetts, and Nevada–experienced increases in prisoner mortality rates between 2000–2004 and 2010–2014. Kansas experienced the largest increases in mortality, 518% in 2010–2014 (as a percentage change from 2000–2004), despite having relatively lower mortality rates across the three periods (see the last column). Maryland, on the other hand, reports that prisoner deaths declined to zero by 2010–2014. The remaining thirty states experienced significant declines in prisoner deaths by 2010–2014, compared to 2000–2004.

The precipitous reduction in prisoner mortality rates during the second decade of the millennium raises questions about the drivers of these declines. We investigate whether certain state-level factors are associated with increases or decreases in state-level prison mortality rates. Table 3 presents estimates from an ordinary least squares regression model of the association between state-level criminal justice and socioeconomic factors on prison mortality rates for states.

**Table 3:** OLS estimates of the association between state-level criminal justice and socioeconomic conditions on prison mortality rates in states reporting to the NCRP, 2000–2014.

	(1)	(2)	(3)	(4)	(5)	(6)
VARIABLES	Baseline	M1 + crime	M2 + Socioeconomics	Baseline w/F.E.	M2 w/F.E.	M3 w/F.E.
Imprisonment Rate	-0.000294	-0.00115	-0.000950	-0.000194	-0.00141	-0.00147
	(0.000663)	(0.000773)	(0.000734)	(0.00114)	(0.000953)	(0.000942)
Violent Crime Rate		0.000801	0.000727		0.00264**	0.00233**
		(0.000576)	(0.000529)		(0.000787)	(0.000820)
Property Crime Rate		0.000228*	0.000229*		7.47e-05	9.16e-05
		(9.21e-05)	(9.20e-05)		(0.000169)	(0.000180)
Democrat Governor			0.0958			-0.0567
			(0.0816)			(0.0820)
Poverty Rate			-0.0189			0.00574
			(0.0249)			(0.0208)
Unemployment Rate			0.0217			-0.0612
			(0.0227)			(0.0488)
Constant	-6.741***	-7.439***	-7.432***	-6.778***	-7.591***	-7.280***
	(0.262)	(0.230)	(0.274)	(0.689)	(0.720)	(0.781)
N (state-years)	534	534	534	534	534	534
R-squared	0.004	0.114	0.124	0.556	0.581	0.586

Authors’ calculations from a OLS regression model predicting the natural log of state mortality rates using data from the NCRP, BJS, UCR, and UKCPR. Models 4–6 include state and year fixed effects (F.E.) to control for state-specific and period-specific events. All robust standard errors have been clustered on state id.

*** p<0.001

** p<0.01

* p<0.05

+ p<0.10.

To expand on Table 3, the imprisonment rate (the baseline regression, Model 1) is not associated with logged mortality rates. However, property crime is associated with increases in prisoner mortality rates (Model 2). In Model 3, the point estimate of the association between property crime on mortality is reported as 0.000229. This is the average estimate of the association across all observations (state-years) in the data file. Because the average property crime rate across all observations (state-years) is 3152.9 (as reported in S1 Table, Supplementary Appendix), the combination of these quantities means that the prison mortality rate predicts or explains 0.72 (= 3152*0.000229) prisoner deaths (in log units) in the average state. The unlogged prison mortality rate associated with property crime means that the property crime rate in the average state is associated with 2.05 (= e(^0.72^)) inmate deaths per 10,000 prisoners. Property and violent crime explain approximately 1/9^th^ of the total variation in state-level prison mortality rates. The political party of the governor, poverty rates, and unemployment rates are not associated with increases or decreases in prisoner mortality rates (Model 3). Including state and year fixed effects in the regressions (Models 4–6) diminishes the association between property crime rates and prisoner mortality rates to statistical insignificance. However, violent crime rates are strongly and positively associated with prisoner mortality rates, after controlling for socioeconomic and political factors (Model 6), suggesting that states with average levels of violent crime increase prisoner mortality rates by 0.89 (= 398*0.00233) log units.

### Mortality decline or disappearing deaths?

Research on mass incarceration demonstrates that incarcerated persons are often excluded from official measures of social inequality [8,113], and there is reason to believe that official death counts reported may be underestimated in the National Corrections Reporting Program (NCRP) [28,29]. Indeed, as Mitchell and Aronson [138] have shown, the criminal legal system systematically underreports deaths in custody, obfuscating one of the more pressing public health issues of our contemporary moment. We explore whether the official death counts reported to the federal government and contained in the NCRP data match official state reports that monitor changes in prisoner death rates [89,116–118]. Our inquiry is motivated by the fact that the precipitous and pronounced decline in prisoner mortality rates may be a statistical artifact of underreporting deaths to the federal government (i.e., that deaths have disappeared from the formal mortality ledger).

**Fig 3:**
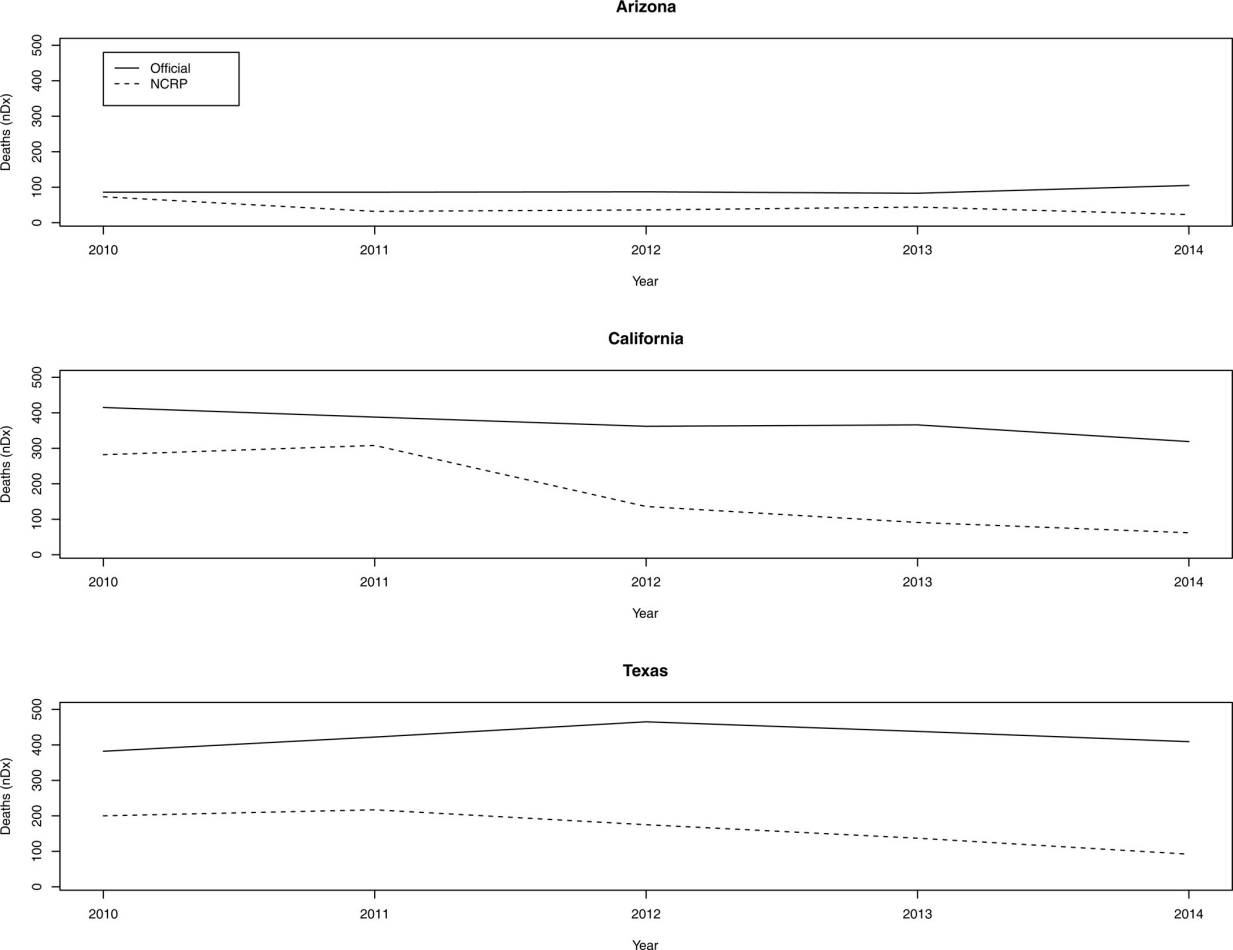
Prisoner death counts in official state reports and the National Corrections Reporting Program (NCRP), 2010–2014. Note: Authors’ calculations from official state reports and the NCRP data.

Fig 3 presents the raw death counts for Arizona, California, and Texas from 2010 through 2014 (the third period in our previous analyses). The solid line represents the trend for prisoner deaths reported in official state documents, and the dashed line tracks the total count of prisoners who died in the NCRP data. There are three main conclusions from these graphs. First, the NCRP significantly undercounts deaths, in comparison to official state reports. Nowhere is this observation more glaring than in California and Texas, where the number of deaths disappeared ranges from a low of 80 (in California during 2011) to a high of 317 (in Texas during 2014).

The second conclusion from this figure is that the systematic underreporting of deaths appears to be increasing in California and Texas and relatively flat in Arizona. The degree to which prisoner deaths disappear from the formal ledger of prison mortality in other states is an open question and may depend on whether those states are under court-order to produce annual death counts. Future research should systematically document deaths in all states that are under some form of legal receivership.

Lastly, it is unclear whether the deaths underreported in Fig 3 are uniformly distributed among incarcerated people, suggesting that the mortality declines observed in previous analyses for particular demographic groups may be an illusion, which would be consistent with the body of literature that documents the disappearance and exclusion of people in prisons and jails from national statistics as a consequence of data collection methods [5,6,8,9,105,113]. Therefore, NCRP estimates of racial disparities in prisoner deaths may be *conservative* if deaths have disappeared from official statistics, contingent on the underlying distribution of mortality rates being lessened by some unknown quantity by race, sex, and age.

## Conclusion and discussion

Mass incarceration in America is a biopolitical institution that structures, at least in part, racial and social inequality [119]. By disappearing entire populations of Black men and women from civil society [8], as well as from national-level demographic data, the prison system performs a state-sanctioned erasure of inequality across a variety of social indicators [8,113,120]. The manufacturing of biopolitical ignorance has the effect of distinguishing those whose life and health are matters of concern from those whose life and health may be disregarded. This problem is further compounded by the prison system’s inadequate healthcare provisions. As such, underreported data present severe obstacles not only for social scientists to provide lawmakers with robust evidence that can accurately identify potential solutions to social problems, but also for policy that seeks to reform and improve prison conditions by enforcing court-mandated compliance orders.

The foregoing analysis shows that the mortality of incarcerated people has dropped in the wake of the Great Recession. While these gains in survivorship translate into unequal increases in life-expectancy by race and sex, recent declines in mortality across states are associated with changes in violent crime. Whether these declines are real is a question open for inquiry, as the distribution of deaths among underreported cases may upend and reverse any notable gains in prisoner life-expectancy. Indeed, one of the contributions of this study is that we have developed a statistical method that may be used most effectively with more reliable data. Although we cannot determine the true trend in prison mortality nationally at this time, the issue of underreporting to which we have demonstrated intersects with several important social and legal issues that should be considered in more detail.

In our analysis of the changing patterns of life expectancy in prisons, we also identify a potential pattern of underreporting within the NCRP dataset, with respect to prisoner death counts. Contrary to official statistics, our findings suggest that prison death counts may be on the rise. In terms of a Foucaultian model of biopolitics, this finding directs our attention to a significant problem in healthcare utilization and implementation. The fundamental utility of biopolitical knowledge is to “provide information on both the effects of, and the consequent necessary corrective changes to be made…” (p. 234) [104] in the governance of life. However, inaccurate government-generated data undermine potential correctives. As we argue, the underreporting of prison mortality fits within broader institutional practices by which the lives of incarcerated people are devalued and rendered unworthy of care or concern by the state.

In the context of our study, the underreporting of mortality inside prison disrupts the capacity to both definitively measure racial disparities within mortality rates and to propose potential solutions. Furthermore, legal statutes and court-mandated compliance orders that govern the prison system—especially in the context of mortality, medical provisions, and healthcare policy—are informed by state-generated reports and investigative bodies that produce data subject to state scrutiny. Thus, underreporting not only intensifies mass incarceration’s role in reproducing racial inequality but also compounds this issue in conjunction with health disparities of which the public is made ignorant [121,138].

The persistence of high institutional mortality rates despite decarceration efforts calls into question the exact relationship between policy change and prisoner survivorship. California is at the epicenter of socio-legal and political reforms aimed at reducing prisoner mortality. California’s prison population significantly expanded beyond its 79,858 capacity, peaking at 173,000 by 2006 [122]. At the height of California’s prison overcrowding crisis, there were 424 deaths reported, at the rate of roughly 248 per 100,000 prisoners [89]. In 2011, the U.S. Supreme Court ruled in *Brown v*. *Plata* that California’s prison overcrowding resulted in cruel and unusual punishment, violating the Eighth Amendment of the U.S. Constitution. As a result, the Court ordered California to reduce its prison population by 33,000 people to 137.5% of design capacity [123].

In response to *Plata*, a series of prison reform measures were set in motion by the state of California in order to reach its population reduction mandate. The first major reform, the “Public Safety Realignment Initiative, AB109” passed in 2011, aimed to: (1) identify low-risk state prisoners to relocate to county jails; (2) shift parole supervision responsibility to county jails while also shortening potential parole periods; and (3) to relax parole revocation proceedings [123,124]. AB109 was followed by two ballot measures which also helped to reduce overcrowding. Passed in 2012, Proposition 36 amended the state’s Three Strikes law to impose a life sentence only when the third felony is “serious or violent,” which also enabled prisoners previously convicted under the law to appeal their sentences [125]. And in 2014, Proposition 47 allowed for certain crimes to be re-classified as misdemeanors rather than felonies, thus softening the potential for long prison sentences [126]. As a result of this period of decarceration and reform, the present-day prison population of California has fallen to roughly 115,000 people. [46].

While the state has reduced its prison population in accordance with the *Plata* decision, our analyses in Table 2 show that prisoner mortality during the 2010–2014 period significantly decreased, even though the relative rank of California in national prisoner mortality remained unchanged. These findings suggest that the national drop in prisoner mortality was uneven over the fifteen-year period for different states. Furthermore, recent research suggests that the gains in survivorship among incarcerated people in California may have been short-lived. A 2019 prison mortality report released by California Corrections Health Care Services (CCHCS) reveals that in 2018, the California Department of Corrections and Rehabilitation (CDCR) experienced a record number of prisoner deaths [96]. During this time there were 452 reported deaths at the rate of 351 per 100,000 prisoners (p. 23–4) [96]. Between 2018 and 2021, the number of reported deaths fluctuated and peaked once again in 2020 at 492 mortalities, while the rate of death per 100,000 continues to trend high [116].

California’s prisoner mortality review process is unique in that it occurs directly under the supervision of a Federal Receivership mandated under the *Plata* court. CCHCS is overseen by J. Clark Kelso, appointed as Federal Receiver by the *Plata* court in 2008, as part of a sweeping effort to reform CDCR’s entire medical care system. The Mortality Review Committee reviews each prisoner death closely by examining the circumstances of death alongside a record of the patient’s medical history [89]. The investigation is used to categorize each death as “expected or unexpected,” with or without “opportunities for improvement,” as a way of tracking systemic lapses in delivering medical care (p. 2–3) [89]. While the stated mission of the Mortality Review Committee is to “reduce avoidable morbidity and mortality and protect public health…” (p. 1) [89] the recent increase in the rate of prisoner deaths in CDCR warrants a more careful investigation into obstacles that frustrate this stated goal. If current reforms conducted under decades of Federal oversight are failing to redress mortality, alternative and supplementary policies should be explored. This could be reason for lawmakers to consider comprehensive sentencing reform, including the elimination of life and long-term sentences, known to significantly contribute to increases in the elderly incarcerated population.

The *Plata* decision also significantly impacted the state of Arizona, where similar healthcare litigation has been pursued against the Arizona Department of Corrections, Rehabilitation, and Reentry (ADCRR). Filed in 2012, the ongoing class-action lawsuit, *Jensen v*. *Thornell* demonstrates systematic failure of the ADCRR to provide adequate medical, mental, and dental healthcare, resulting in unnecessary sickness, injury, and death of prisoners [127]. ADCRR contracts its healthcare services to for-profit vendors. One of the central outcomes of the Jensen litigation ‐ during the time period at interest for this study ‐ was the reporting and monitoring of 103 healthcare performance measures to be implemented by the ADCRR [128]. ADCRR continuously failed to satisfy the requirements set by the performance measures, leading to contempt of court orders, multiples fines of over $1 million dollars, and the eventual rescission of the performance measures and imposition of a permanent injunction. One of the court’s contempt orders cited 18 mortality review reports as evidence of failure to meet proper healthcare standards. “ADC checked ‘yes’ 6 times to the question: ‘Could the patient’s death have been prevented or delayed by more timely intervention.’ ADC checked ‘yes’ 8 times to the question: ‘Is it likely that the patient’s death was caused by or affected in a negative manner by health care personnel.’” (p7-8) [129]. Moreover, a mandated expert report emphasized that privatized healthcare will continue to pose a barrier to compliance due to increased costs and the institutional challenges and delays that come with vendor oversight. Mortality reviews are of particular importance here as the expert report found significant issues in the identification and remediation of organizational errors that have contributed to prison death. The report concluded that ADCRR should return to operating its own healthcare services [130].

The situation in California and Arizona highlights not only the issue of providing constitutionally adequate healthcare in prison, but illustrates a potential mechanisms by which prison deaths are produced. Unlike California, where the prison healthcare system is under federal receivership and has received extensive financial investment from the state, the Arizona prisoner healthcare system is privatized and under resourced [131]. Despite differences in their healthcare management and supervision, both states have exhibited systematic errors in conducting mortality reviews, as well as formalizing appropriate remediation plans to prevent similar situations from taking place [17,127,130–132]. Such institutional shortcomings might help explain and explore the upticks in prison mortality. At the very least, increases in prisoner mortality, as well as their underreporting, raise questions about the effectiveness of healthcare interventions inside carceral institutions and suggests that wholescale structural changes may be necessary to stem the tide of prison death.

While the cases of California and Arizona demonstrate the lethal failures of prison health care, the social determinants of health, which precede an individual’s incarceration, yet are deeply entangled with mass incarceration, also influence prison mortality rates. While the health profiles of inmates are especially dire, incarcerated people are one of the few classes of people with a constitutional right to healthcare. However, the implementation and delivery of prison health services is far less than adequate. Historically, attempts at standardized care, accreditation, and provider professionalization have consistently been undercut by institutional priorities of coercion and control [133–136]. In fact, one study of an Alabama prison medical ward demonstrates how prison healthcare provisions are so abysmal that medical neglect has become a normalized aspect of incarceration [20]. Moreover, institutional variation in the delivery of prison medical services makes it increasingly difficult to pinpoint the precise mechanisms that result in such poor health delivery outcomes [31]. Health care is typically managed by either state department, private vendors, or university medical school systems. Still, improved care can reduce mortality rates. A study of inmate mortality in the Texas prison system–managed by the state’s medical school system ‐ concluded that deaths declined overall due to meaningful improvements in the management protocols for non-infectious diseases, implementation of performance evaluations, and the organization of oncology clinics and geriatric units [54]. And while the ACA has offered state prisons a potential healthcare cost incentive, it remains largely underutilized [137–140]. Incarcerated people are excluded from Medicaid while they are incarcerated, but the cost of inpatient hospitalization that lasts more than 24 hours may be reimbursed by Medicaid for eligible prisoners. However, this incentive is unavailable to those states that have opted out of Medicaid’s expansion of adult eligibility.

While we have endeavored to explore how mortality has changed prior to the COVID-19 pandemic, as well as the factors and processes affecting this life event, there are several limitations to our study. First, although the NCRP provides “Hispanic/Non-Hispanic” ethnic distinctions in their data, if states did not collect this information at the time a person was imprisoned prior to the collection of the NCRP data, then trends for Hispanics may be underestimated while trends for Non-Hispanic racial groups may be overestimated. The extent to which there is over- or under-estimation may be based on 1) when each state began collecting ethnicity and 2) the racial distribution of people who are Hispanic and Non-Hispanic in a state’s prison system. Unfortunately, it is unknown when each state began collecting ethnicity as a race-based measure.

Second, we do not have specific measures of the quality of healthcare associated with each state’s prison system. Such measures, as well as the quantity of healthcare staff performing testing and treatment, would assist in pinpointing additional structural factors that affect the health and mortality of people in prison.

Third, the NCRP data do not provide measures of people’s health before or during confinement. Thus, it is possible that health selection effects among people imprisoned may drive differences in mortality between demographic groups. We are unable to examine this proposition; however, our focus is not on how preexisting health differences cause mortality, but rather on how measures of mortality have changed over time for specific demographic groups of people imprisoned; how state-level factors may increment or decrement mortality levels; and how the very measurement of mortality itself may be underestimated when benchmarked to other data sources. Thus, policy determinants of who gets imprisoned and their overall health, and the corresponding effects of health selection into prison on differences in prison mortality, should be studied in future research.

Finally, while our study cross-validates mortality counts in a few states known for healthcare concerns, future research should document and cross-validate all NCRP data for mortality undercounting. It could be that some states are better or worse in enumerating death in custody than the states in our study. Such an endeavor will require significant resources to identify independent reports produced by state and federal investigators in order to assess the degree to which mortality may be underestimated throughout state prison systems in the United States.

Ultimately, the promises and perils of criminal justice reform are reflected in prisoner mortality rates. Legal decisions and social policies aimed at reducing mortality may be most effective in the short-run; however, the effects of these policy changes may fadeout over time. Understanding how and why gains in survivorship may stall is important for aligning health initiatives with social policy to facilitate maximal and consistent mortality declines for all demographic groups.

## Supporting information

S1 Table State-level descriptive statistics for conceptual measures and control variables, selected states 2000. (DOCX)
