# Supplementary material for: Death and disappearance: Measuring racial disparities in mortality and life expectancy among people in state prisons, United States 2000–2014
Source: PLoS One. 2025 Feb 6;20(2):e0314197. doi: 10.1371/journal.pone.0314197 (PMC11801571; doi:10.1371/journal.pone.0314197)
Supplement: S1 Table — (DOCX) [file pone.0314197.s001.docx]

**Supplementary Appendix Table (S1): State-Level Descriptive Statistics for Conceptual Measures and Control Variables, Selected States 2000-2014**

| *Variables* | *Operationalization* | *Coding* | *Mean* | *SD* | *Min.* | *Max* | *N* |
| --- | --- | --- | --- | --- | --- | --- | --- |
| *Dependent Variables* |  |  |  |  |  |  |  |
| Mortality Rate (nMx) | Ratio of deaths in prison relative to the prison population | Continuous | 0.0010 | 0.0008 | 0 | 0.0032 | 635 |
| ln(Mortality Rate (nMx)) | ln(Ratio of deaths in prison relative to the prison population) | Continuous | -6.865 | 0.692 | -9.95 | -5.76 | 534 |
| *Independent Variables* |  |  |  |  |  |  |  |
| Imprisonment Rate | Number of state prisoners per 100,000 residents | Continuous | 416.4 | 159.9 | 126.0 | 881.0 | 635 |
| Violent Crime Rate | Number of violent offenses per 100,000 residents | Continuous | 397.9 | 163.2 | 78.2 | 828.1 | 635 |
| Property Crime Rate | Number of property offenses per 100,000 residents | Continuous | 3152.9 | 779.0 | 1718.2 | 5850 | 635 |
| Governor Democrat | Governor of the State is Democrat | Yes = 1, No = 0 | 0.47 | 0.5 | 0 | 1 | 635 |
| Poverty Rate | State poverty rate (percent) | Continuous | 12.7 | 3.3 | 4.5 | 23.1 | 635 |
| Unemployment Rate | State unemployment rate (percent) | Continuous | 6.0 | 2.1 | 2.5 | 13.7 | 635 |
